# Chair based exercise in community settings: a cluster randomised feasibility study

**DOI:** 10.1186/s12877-018-0769-4

**Published:** 2018-04-03

**Authors:** K. R. Robinson, A. L. Long, P. Leighton, S. Armstrong, R. Pulikottill-Jacob, J. R. F. Gladman, A. L. Gordon, P. Logan, K. A. Anthony, R. H. Harwood, P. E. Blackshaw, T. Masud

**Affiliations:** 1 Division of Rehabilitation and Ageing. Medical School, University of Nottingham, Nottingham, UK; 2 Duncan McMillan House, Nottinghamshire Healthcare NHS Foundation Trust, Nottingham, UK; 3 Division of Medical Sciences and Graduate Entry Medicine, University of Nottingham, Nottingham, UK; 4 School of Medicine, University of Nottingham, Nottingham, UK; 5 Clinical Trials Unit, University of Warwick, Coventry, UK; 6 National Institute for Health Research (NIHR) Collaboration for Leadership in Applied Health Research and Care East Midlands (NIHR CLAHRC EM), Nottingham, UK; 7 Nottingham Biomedical Research Centre (BRC): Musculoskeletal Disease (MSK) theme, Nottingham, UK; 8 Healthcare of Older People, Queens Medical Centre, Nottingham University Hospitals NHS Trust, Nottingham, UK; 9 Medical Physics, Queens Medical Centre, Nottingham University Hospitals NHS Trust, Nottingham, UK

**Keywords:** Older people, Exercise, Care homes

## Abstract

**Background:**

Some older people who find standard exercise programmes too strenuous may be encouraged to exercise while remaining seated - chair based exercises (CBE). We previously developed a consensus CBE programme (CCBE) following a modified Delphi process. We firstly needed to test the feasibility and acceptability of this treatment approach and explore how best to evaluate it before undertaking a definitive trial.

**Methods:**

A feasibility study with a cluster randomised controlled trial component was undertaken to 1. Examine the acceptability, feasibility and tolerability of the intervention and 2. Assess the feasibility of running a trial across 12 community settings (4 day centres, 4 care homes, 4 community groups). Centres were randomised to either CCBE, group reminiscence or usual care. Outcomes were collected to assess the feasibility of the trial parameters: level of recruitment interest and eligibility, randomisation, adverse events, retention, completion of health outcomes, missing data and delivery of the CCBE. Semi- structured interviews were conducted with participants and care staff following the intervention to explore acceptability.

**Results:**

48% (89 out of 184 contacted) of eligible centres were interested in participating with 12 recruited purposively. 73% (94) of the 128 older people screened consented to take part with 83 older people then randomised following mobility testing. Recruitment required greater staffing levels and resources due to 49% of participants requiring a consultee declaration. There was a high dropout rate (40%) primarily due to participants no longer attending the centres. The CCBE intervention was delivered once a week in day centres and community groups and twice a week in care homes. Older people and care staff found the CCBE intervention largely acceptable.

**Conclusion:**

There was a good level of interest from centres and older people and the CCBE intervention was largely welcomed. The trial design and governance procedures would need to be revised to maximise recruitment and retention. If the motivation for a future trial is physical health then this study has identified that further work to develop the CCBE delivery model is warranted to ensure it can be delivered at a frequency to elicit physiological change. If the motivation for a future trial is psychological outcomes then this study has identified that the current delivery model is feasible.

**Trial registration:**

ISRCTN27271501. Date registered: 30/01/2018.

**Electronic supplementary material:**

The online version of this article (10.1186/s12877-018-0769-4) contains supplementary material, which is available to authorized users.

## Background

Muscle strengthening and balance exercise programmes that involve exercising when standing are widely employed in clinical practice [1]. These programmes have been shown to reduce the risk of falls with an associated impact on mortality, morbidity and costs to health and social care [2]. Declining health and physical limitations may however prevent some older people from taking part in these well evidenced standing programmes. Pragmatic approaches have evolved where exercise is performed primarily in the seated position- chair based exercise (CBE). Such CBE programmes are now commonly delivered across health and social care to older adults [3] with compromised health and mobility.

A systematic review of the physical benefits of CBE for frail older people [4] found little rigorous CBE research, with little consensus about treatment, or whether it has any benefits. The review acknowledged the difficulties of identifying relevant literature and a lack of a clear understanding surrounding CBE as an intervention. To develop a better understanding of CBE a modified Delphi consensus process was previously undertaken with experts [5]. The Delphi panel experts agreed that CBE should contain components of progressive resistance training, cardiovascular interval training, endurance training and developmental stretches with the aim of improving mood and well-being, muscle strength, activities of daily living and joint mobility. The experts also identified that CBE should be used for older people who are unable to take part in other forms of exercise due to activity limitation which may be acute (e.g. following an operation) or longer term, and should be undertaken at least once a week. This consensus chair based exercise (CCBE) is a complex intervention as it involves a number of interacting components, it is delivered across different settings and has multiple outcomes of interest [6]. In line with the Medical Research Council guidance for evaluating complex interventions feasibility work was indicated prior to evaluating the newly defined CBE intervention in a definitive trial [7]. We firstly needed to test the feasibility and acceptability of this CCBE intervention and explore how best to evaluate it before undertaking a definitive trial.

In this study we aimed to examine the acceptability, feasibility and tolerability of the CCBE intervention as well as the feasibility of running a cluster randomised controlled trial (RCT), exploring appropriate outcomes and ascertaining data for economic evaluation.

## Methods

A multi-centre, three armed, feasibility cluster randomised controlled trial with blinded outcome assessment was undertaken across community settings in Nottinghamshire. The three arms were: the CCBE intervention, an active control (group reminiscence) and usual care. Ethical approval was provided by the National Research Ethics Service Committee Nottingham One (Reference: 15/EM/0005).

### Recruitment and settings

#### Recruitment of centres

Recruitment of centres providing services for older people took place between February 2015 and July 2015. Three main types of centre were identified where CBE might already be delivered: day centres, care homes and voluntary run community groups (Table 1). Potential centres were approached by letter and a follow up telephone call and invited to express an interest in taking part in the study. From those that expressed an interest twelve were selected using a purposive sampling method to ensure the sample included four day centres, four care homes, four community groups and covered a range of demographics (e.g. size of centre, type of centre, funding source). For care homes we aimed to select two residential homes, one dementia registered home and one non-dementia registered home.

**Table 1:** Types of centre

Type of centre	Definition	Sources used to identify centres
Care Homes	Institutions providing accommodation and 24 h care for older people, with or without nursing care	Care Quality Commission database
Day Centres	Local authority run centre that provided day services for older people	Local authority database
Community groups	Charitable/voluntary run service that provided day activities for older people	Social work department and signposting agencies (such as Age UK, Alzheimer’s Society and The Parkinson Society)

#### Older people

Once four day centres, four care homes and four community groups had been selected staff at all centres were asked to identify eligible older people who were 65 years and over, required assistance for walking and were able to understand and follow instructions. Older people who had the mental capacity provided their own consent. Those residents without mental capacity were recruited following a family member or friend completing a consultee declaration to indicate that the research was not contrary to their wishes (in accordance with the English Mental Capacity Act 2005). Following consent mobility measures were completed to determine whether participants were appropriate for the CCBE intervention as defined in the consensus study [5]. These measures were the Timed Up and Go Test (eligible if scoring 20 s or more) [8] and a four metre walk test (eligible with a gait speed of 0.6 m/s or less [9]).

### Randomisation

Randomisation was undertaken at the centre level and stratified by the type of centre (day centre, care home and community group). Using statistical software STATA, random numbers in the range 0–2 were generated and attached to each centre ID. Randomisation was completed by an independent statistician with the allocation given to the trial manager and the teams delivering the interventions.

### Intervention

#### CCBE intervention

The CCBE intervention was delivered by a research physiotherapist in six centres (two day centres, two care homes and two community groups) in a group with the aim of participants engaging a minimum of once a week (twice weekly if able) for twelve weeks and with each session lasting an hour as defined in the consensus study [5]. Each session included: warm up, progressive strength resistance training, cardiovascular interval training, endurance training delivered at a moderate intensity, developmental stretches and cool down. Music was offered and used if it was welcomed by the group. The exercises were adapted (the number or sets, the number of repetitions, the level of resistance) by the physiotherapist to meet the needs of each individual participant to account for the differences between participants.

#### Group reminiscence

Group reminiscence therapy was delivered once a week for twelve weeks by a trained Age UK staff member. This intervention was the active control, providing group support and social activities without exercise. The feasibility and acceptability needed to be established prior to a definitive trial and to ensure it could be fully described.

#### Usual care

Centres continued as usual without any involvement from the study team. This arm was needed to establish whether usual care would be a feasible control in a definitive study if it was found that group reminiscence could not be used as a control.

### Feasibility of the trial parameters

The outcomes collected to assess the feasibility of the trial parameters were: the level of recruitment interest and eligibility, randomisation, adverse events, retention, completion of health outcomes, missing data and delivery of the CCBE intervention and group reminiscence. Data were stored in a Microsoft Access Database and analysed using the statistical package SPSS 23. Descriptive statistics were used to summarise the outcomes for the trial parameters.

#### Level of interest and eligibility

The number and percentage of eligible hosts (day centres, care homes and community groups) identified and the number of centres that expressed an interest in participating was recorded.

#### Randomisation

The willingness of centres to be randomised and any drop out of centres due to allocation was recorded.

#### Group characteristics

Characteristics of participants in each setting (day centres, care homes and community groups) were collected to determine if participants across the different types of centres were sufficiently similar to be studied together. These were: age, gender, diagnosis of dementia, level of dependence (Barthel Index) and level of functional mobility (Timed Up and Go Test).

#### Adverse events

The following adverse events which were considered could be related to the CCBE intervention and group reminiscence sessions were collected: falls, fractures, cardiac chest pain, head injuries, pain and emotional distress.

#### Retention

The number of participants who withdrew from the study at which stage and the reason for withdrawal was recorded.

#### Completion of health outcomes

A researcher blinded to the allocation collected data at baseline and three and six months after randomisation. Outcomes included; grip strength using a Jamar hand held dynamometer [10], lower limb muscle strength using the 30 s chair stand [11], well-being using the Warwick Edinburgh Well-Being Scale [12, 13] (all centres), Social Well-being of Nursing home scale [14] (care homes only) and cost analysis using Client service Receipt Inventory (CSIR) questionnaire and health related quality of life analysis using EQ-5D-5 L [15]. The 30 s chair stand test was added as a measure to trial and was therefore only completed in the community group cohort.

#### Delivery of interventions

CCBE and group reminiscence attendance rates were recorded along with reasons for non-attendance. The number of sessions delivered per week and barriers to delivery were recorded. Field notes were kept by the researchers, physiotherapist and Age UK staff on the delivery of the CCBE intervention, delivery of the group reminiscence and the trial processes.

### Embedded qualitative study

Participants who had taken part in the CCBE programme and who had capacity to provide consent were invited to take part in an semi-structured interview about their experiences of the programme and the research study design. The interviews took place at the centre where the participant attended. Staff at the centres were invited to take part in a one-off interview to discuss their views on the research study and exercise interventions in these settings.

Interviews were managed to last no longer than one hour (and in some cases were significantly shorter); data were captured using digital audio recording equipment and transcribed in full. The semi-structured interview schedule was developed to consider experience of the CCBE programme, facilitators and inhibitors to participating in CCBE, improving CCBE, participating in the research process.

Analysis used framework analysis; a hierarchical, matrix based method developed for applied research which allowed focused interrogation of data in a relatively short space of time [16]. Data were coded within a pre-defined thematic framework (determined by current literature and the team’s clinical knowledge) which was structured to consider the perception of CBE, programme content, benefits, barriers and facilitators to participation. To allow for any views expressed through the interviews that did not fit with the pre-defined framework an additional code of ‘other’ was added to ensure this data was not missed. It was acknowledged that this framework could be adapted in the context of the data through the creation and removal of codes however the original framework and any adaptations would be clearly reported and justified by the data. The process of analysis followed the stages of coding, charting and summarising. Interview data were managed using NVIVO (Version 10.0) data management software.

## Results

**Fig. 1:**
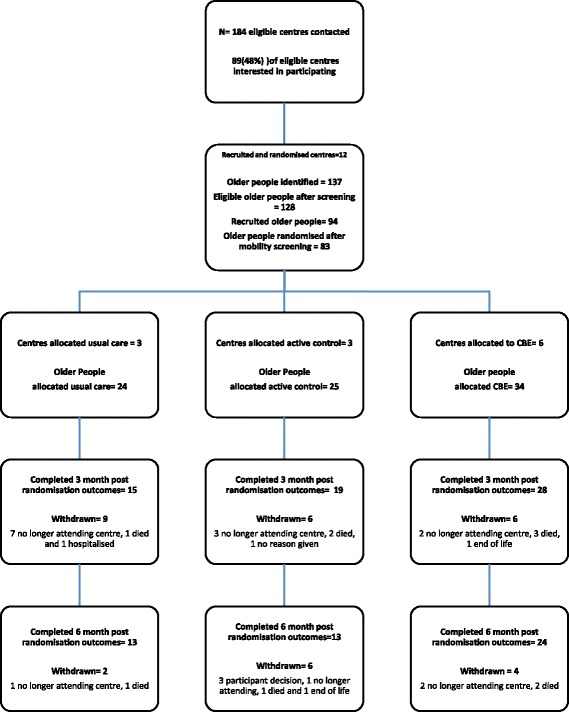
Overview of participants through the study

Figure 1 provides an overview of the flow of centres and participants in the study.

Semi-structured interviews were conducted with eight older people (one day centre, three care home residents and four community group attendees), twelve care staff (four day centre, six care home, two community group) and a telephone interview with one consultee. Findings from the interviews, questionnaires, and field notes presented together to address the feasibility objectives. Further information is provided in the Additional file 1: Tables S3 and S4.

### Level of interest and eligibility

One hundred eighty-four centres were contacted with 89 (48%) expressing an interest in taking part. 83% of day centres, 45% of care homes and 20% of community groups that were invited interested in participating in the study. The centres had a range of 10–15 older people attending on the selected days when the study was conducted. In the selected twelve centres, 40 service users in the day centres, 45 residents in the care homes and 52 service user in community groups were eligible to take part, with 128 (93%) of these eligible after review by a researcher and 94 (73%) of eligible older people consenting to take part across all centres. There was a higher proportion of eligible participants who consented to take part in day centres (86%) compared to care homes (*n* = 70%) and community groups (67%). 88% (83 participants) of the 94 participants that consented for assessment were eligible after completing the mobility scores and this percentage was similar across centre types.

Recruitment of centres and older people required greater staffing resources (three researchers) than the originally planned single researcher. Additional time and resources were needed to liaise with family and friends to gain consultee declarations (46 participants (49%)) leading to an additional 45 h of staff time. Researcher maintained field notes identified the following recruitment issues; closures of day centres during the recruitment phase of the study due to local funding mechanism, limited time to recruit in each centre due to the existing routine and overly complex governance processes for the level of study and type of participants involved.

The interviews indicated that most staff felt that appropriate older people had participated in the CCBE programme, however there was some concern over using the Timed Up and Go Test to identify eligibility as they reported that the scores were influenced by the time of day. There was disagreement between some staff who felt CBE was the only exercise the participants could do (due their physical characteristics) and the participants who felt they wanted to try more ‘proper’ exercise in standing.

### Randomisation

Twelve centres were randomised and there were no drop out of centres due to allocation.

### Group characteristics

The mean age of the sample was 84 years with 66% being female and 60% having a diagnosis of dementia (Table 2).

**Table 2:** Characteristics of participants

		Total Sample (*n* = 83)	Day Centres (*n* = 28)	Care Homes (*n* = 27)	Community Groups (*n* = 28)
Age (years)	Mean (SD)	84 (9.0)	83 (8.2)	86 (9.0)	83 (9.6)
	Median (IQR)	85 (76–91)	84 (77–90)	87 (78–93)	84 (74–89)
	Range	65–103	68–94	69–102	65–103
Female	N (%)	55 (66.3%)	19 (67.9%)	19 (70.4%)	17 (60.7%)
Dementia Diagnosis	N (%)	50 (60.2%)	16 (57.1%)	15 (55.6%)	19 (70.0%)
Number of prescribed medications	Mean (SD)	6.9 (3.4)	6.2 (3.6)	7.4 (3.7)	7.0 (2.9)
	Median (IQR)	7 (4–9)	6 (3–8)	8 (4–10)	7 (5–9)
	Min to max	0–16	0–16	0–14	1–12
Timed Up and go (seconds)	Mean (SD)	38.2 (24.2)	30.4 (11.4)	41.4 (26.2)	43. 8 (30.4)
	Median (IQR)	30.5 (22.9–46.1)	29.2 (21.7–36.2)	34.7 (24.0–50.69)	34.3 (24.9–50.9)
	Min to max	15.8–145.2	15.8–59.9	19.3–117.1	19.1–145.2
Activities of daily living (Barthel) Score out of 100	Mean (SD)	72.4 (20.1)	77.0 (18.7)	68.7 (17.6)	71.5 (23.3)
	Median (IQR)	75 (60–90)	80.0 (70–95)	70.00 (50–85)	80.00 (60–90)
	Min to max	20–100	20–100	35–100	20–100

### Adverse events

There were no serious adverse events reported that were considered to be related to either the CCBE or the group reminiscence sessions in the day centres or community groups. One event (angina attack) was retrospectively reported by a participant in a care home, however, this was considered not serious and the causality could not be established due to a lack of information from the participant and care home.

Staff largely considered CCBE to be an appropriate and safe intervention. One was however concerned that the programme had been too intense and it was ‘*too late’* in life for some of the participants to be participating in exercise programmes.

### Retention

40% (*n*- = 33) of participants withdrew from the study, ten from the intervention group (five deaths, four no longer attending and one due to poorer health), twelve from the reminiscence group (three deaths, four no longer attending, four participant decision and one due to poor health) and eleven from the usual care group (two deaths, eight no longer attending and one due to poor health).

### Completion of health outcomes

#### Upper limb strength

Eighty-two (99%) of the sample completed the grip strength test at baseline with one participant declining to complete the measure. At the post intervention follow-up two (out of 62 remaining participants, 3%) declined to complete their grip strength. At the six month follow up two (out of 50 remaining participants, 4%) declined to complete their grip strength.

#### Lower limb muscle strength

Fifteen participants (out of 28, 54%) were unable to complete this outcome at baseline. At the post intervention eight participants (out of 19, 42%) were unable to complete the 30 s chair stand. At the six month follow up six (out of 15, 40%) were unable to complete this measure and one participant declined to complete the measure.

#### Well-being

The Warwick Edinburgh Well Being measure was fully completed by 79 participants (out of 83, 95%) at baseline and two participants (out of 62 remaining participants, 5%) declined to complete the measure at the post intervention follow up. At the six month follow up one participant (out of 50 remaining participants, 2%) was unable and four participants (8%) declined to complete this questionnaire.

The Social Well-being of nursing home residents was completed for all care home residents (*n* = 27) at baseline and post intervention follow ups and was completed from the perspective of the care staff.

#### Resource use

Complete data on resource use was available for 82 participants (99%) at baseline and 47 participants (98%) across all data collection points. The questionnaires were completed by the participants (55%), consultees (42%) and care home staff where appropriate (3%).

#### Quality of life

Complete data on the EQ-5D-5 L were available for 81 participants (98%) at baseline and 44 (92%) across all data collection points. The General Health Questionnaire (GHQ-12) was completed by 79 (96%) of the sample at baseline with 2% declining to complete and 2% not being able to complete the questionnaire. At post intervention follow up 94% of the participants remaining in the study completed the GHQ-12. At the six month follow up two participants (out of the remaining 50, 4%) were unable and two participants (4%) declined to complete the GHQ-12.

### Delivery of CCBE

The CCBE was delivered once a week, in a group format, in the day centres and community groups. Support with staff and the provision of transport was offered to facilitate delivery twice a week, however it was not possible to deliver more sessions. Reasons for being unable to facilitate delivery for an additional session were: no available space at the centres, and willingness of participants to attend for an additional session. The CCBE could be delivered twice a week as a group format in care homes; however there were a reduced number of available sessions in one care home due a sewage leak leading to a temporary home closure.

Attendance logs for the sessions held once a week in the day centres (90%) and community groups (71%) demonstrated that no participant attended all sessions. In the care home setting where sessions were offered twice a week there was low attendance with only 48% of available sessions attended. Exercise progression was feasible for participants who attended regularly from the physiotherapist maintained field notes. Participant interviews and physiotherapist field notes indicated that hand weights were preferred to resistance bands for ease of use.

Most participants found the CCBE programme acceptable and reported enjoying taking part, especially the social benefits of a group activity as well as having something to look forward to and participate in. Participants reported in the interviews wanting to try more standing and walking exercise, although some were not confident they would have been able to undertake it.

### Delivery of active control

The group reminiscence was delivered once a week for ten weeks in one care home, one day centre and community group. Difficulty achieving the intended twelve week programme was identified by Age UK Nottinghamshire staff who facilitated the sessions. Reasons included the schedule of the centres, seasonal activities (e.g. Christmas) and their other work commitments which reduced flexibility. Time to plan and effectively resource the sessions within existing work commitments was raised as a challenge which would need to be considered by a future trial. Age UK staff and centre staff reported the reminiscence sessions had been enjoyable and welcomed by older people.

## Discussion

### Summary of findings

The study aimed to establish whether it was feasible to run a cluster RCT across day centres, community groups and care homes. Results neither support nor refute the effectiveness of the CCBE intervention as this was not the purpose of the trial. Although recruitment was challenging and required more staff than was originally anticipated, centres and older people were interested in taking part in the study and it was possible to recruit centres and older people to participate in the trial. The Timed Up and Go Test and gait speed markers appeared to identify older people with activity limitations who were appropriate for the CCBE intervention. Forty percent of participants withdrew from the study primarily due to stopping attending the centres indicating the trial design would need to be revised to follow up participants at home. Outcomes of well-being, grip strength, quality of life and resource could be collected. Further exploration is required to establish the most appropriate lower limb outcome for this population.

This feasibility study also explored the acceptability and feasibility of delivering the CCBE intervention. In the day centres and community groups the CCBE could be delivered in a group format once a week (in line with the expert consensus) and twice a week in care homes. The CCBE intervention was largely accepted by older people and staff at the centres. Older people focused on the social benefits of group activity and having something to look forward to and take part in. Older people also wanted to try more standing and walking exercise but were not confident in their ability to do so. Staff reported the CCBE was appropriate and enabled safety of older people with physical limitations.

This feasibility study has established areas where a future trial design would need to be revised to maximise retention of participants as well as maximise delivery of the CBBE intervention.

### Strength and limitations

The main limitation of this study is that we have tested the feasibility of a particular planned trial, using our version of CCBE, to a specific patient group and with the specific attention control of group reminiscence. There may be different patient groups, different versions of CBE and different settings that have not been included here and the findings of this study may have limited generalisability in those contexts. This study has however met its objective of addressing the feasibility of the CCBE intervention and trial design.

The views of the older people captured through semi-structured interviews are from those who were allocated to the CCBE group and may not be representative of all older people. Due to capacity it was not possible to interview all the older people who took part in the control groups or those older people not eligible to take part in the study, however, their views may have offered further insight into appropriate exercise interventions in these settings. The lack of recruitment to consultee interviews limits the findings of this component of the study and further work is needed to explore this perspective.

### Implications

This study has explored the delivery of the CCBE intervention in complex environments and it is important to acknowledge the difficulties of conducting research and delivering interventions in these settings.

The CCBE intervention could be delivered once a week in the day centres and community groups which was supported by the expert consensus which stated sessions should be delivered at least once a week [5] This frequency of delivery is however not supported by the wider exercise literature for frail older people which indicates higher frequencies are needed to elicit physiological change [17, 18]. Previous work in care home residents has identified issues with delivering exercise interventions at appropriate frequencies with Chin a Paw [19] concluding that exercise programmes that are delivered less than twice a week are not sufficient to elicit functional gains, however supporting participation twice a week was challenging.

The CCBE in this study was delivered in a group format at centres with existing infrastructure and where older people already attended. Using these established centres compromised the delivery of the CCBE at higher frequencies and further work is needed to explore alternative delivery formats. One-to-one sessions offered at participant’s homes, setting up additional community group as well as at the existing centres may facilitate increased frequencies. For a future trial where physical outcomes are targeted the delivery model of CCBE would need to be revised to ensure delivery at a minimum of twice a week. This delivery model would require more resources (therapists, equipment) and the cost implications would need to be considered by the trial evaluation.

As the CCBE intervention was intended for those older people with compromised health and mobility it is unsurprising that health status was a barrier to engagement and progression of the exercise programme. This supports the wider views of older people who identify physical ailments as a potential barrier to long-term exercise engagement [20]. Interventions may need a high degree of tailoring to account for individual health conditions and preferences of older people. Overly structured and prescriptive programmes that do not allow for changes to the delivery may be limited for this population. With this degree of flexibility and variation in delivery it is important that the CCBE intervention is delivered by a professional who has experience and skills to meet the needs of the older people in this study.

Older people enjoyed participating in the CBE programme, however, some participants expressed a preference to progressing to standing and walking if they are able. Progressing to supported and unsupported standing exercise was also considered valuable by experts [5]. Other exercise programmes developed for older people have used CBE as the starting point for participants with poor mobility [21] however there is a lack of detail over whether participants progressed to supported and free standing exercises within these studies. Progression to supported standing exercises following a chair based programme was found to be achievable in a small feasibility study with community dwelling older people [22]. Further work is needed to develop the CCBE intervention to actively support progression to standing and walking programmes if this is achievable by the participant.

At once a week it may be reasonable to consider the CCBE intervention as a way of promoting general well-being with limited influence on physical measures of muscle strength and mobility. This study demonstrated that it was feasible for a future trial to focus on psychological aspects such as well-being and quality of life and this was supported by the views of the older people who participated. Previous research such as the large (*n* = 1054) OPERA trial [23] has evaluated a range of physical activity interventions in care home residents and concluded that there was no effect on mood and depression. This trial suggested that measuring well-being may be more appropriate for exercise interventions and we found that the Warwick Edinburgh Well-Being Scale was able to be completed in this study. A trial of CCBE with the primary focus on well-being could be delivered once a week in a group format and for this outcome it would be appropriate to compare it with an active control. Group reminiscence was demonstrated to be an acceptable active control for this population and has been used successfully in previous care home research [24].

Although there has been an increase in research conducted in care homes [25], the other types of centres in this study (day centres and community groups) have not frequently been exposed or involved in clinical research. It was encouraging that these centres were interested in taking part in research that can support older people as the services for older people are increasingly being supported by the third sector. Non-NHS sites fall outside of traditional governance frameworks and time needs to be planned to obtain agreements from each centres taking part. Traditional participant information sheets for clinical trials are often expected by ethical committees and research governance teams and these can be a deterrent to recruitment for studies in these settings. There is a need to consider the study processes and information to ensure they meet the needs of the participants and settings. Consideration of appropriate governance procedures and information that is appropriate to the setting would be needed to maximise recruitment and reduce the burden to centre staff and participants.

The process of randomisation was explored in this study in preparation for a definitive randomised controlled trial. Although there was no drop out of centres due to allocation some centres who were allocated to receive usual care expressed concern over the burden of the research for little immediate gain. Given the propensity for bias in this field, we argue that the most robust information about effectiveness will come from high quality RCTs and therefore considered an RCT to be the most appropriate form of definitive evaluation for the CBE intervention. The high dropout rate of participants in this study, the flexible delivery and the difficulties engaging centres in the control arm may however suggest alternative evaluation methods. Recruitment and follow up of participants should include home based assessments to allow follow up of participants who no longer attend the centre and to ensure minimal disruption to centre activities.

## Conclusions

This study was designed to establish whether a definitive trial was justified and avoid an expensive large trial that was not able to address the research aims. There was a high level of interest from community centres and older people for this research and the CCBE intervention was largely welcomed. The trial design and governance procedures would need to be revised to maximise participant recruitment and retention. The primary focus on physical or mental health will dictate the direction of a future trial.

If the motivation for a future trial is physical health then this study has identified that further work to develop the CCBE delivery model is warranted to ensure it can be delivered at a minimum frequency to elicit physiological change. If the motivation for a future trial is psychological outcomes then this study has identified that the current delivery model is feasible.

## Additional file

Additional file 1: **Table S3.** Summary views of older people. **Table S4.** Summary views of staff. Views of older people and staff on the CCBE intervention from the qualitative interviews. (DOCX 19 kb)

## Abbreviations

CBE: Chair based exercise
CCBE: Consensus chair based exercise
RCT: Randomised controlled trial
