# Supplementary material for: Chair based exercise in community settings: a cluster randomised feasibility study
Source: BMC Geriatr. 2018 Apr 3;18:82. doi: 10.1186/s12877-018-0769-4 (PMC5883353; doi:10.1186/s12877-018-0769-4)
Supplement: Supplementary file 1 — Table S3. Summary views of older people. Table S4. Summary views of staff. Views of older people and staff on the CCBE intervention from the qualitative interviews. (DOCX 19 kb) [file 12877_2018_769_MOESM1_ESM.docx]

**Additional file 1**

Table 3: Summary of views of older people

|  | Summary | Supporting quotes |
| --- | --- | --- |
| Perceptions of CBE | Generally enjoyed taking part and doing something different. Recognised the use of CBE as appropriate due to difficulties with standing and health. There was a feeling of wanting to progress to more standing and walking. | *‘The reason I say it is very good is because I had a disability of I cannot use my legs at all’’* |
|  |  | *'I loved it'* |
|  |  | *‘I always think I could do more- proper standing up exercise, but whether or not I could that is different you know’* |
|  |  | *‘Well disappointed’ [in response to no walking in programme]* |
| Benefits of CBE | The main reported benefits related to well-being and enjoyment from taking part. Benefits relating to improved ease of movement and mobility were also reported. There were few negatives reported, however, participants did notice an ache after taking part. | *‘…used to have this click, click, click and now it don't click at all’[shoulder]* |
|  |  | *‘I couldn't wash my neck and I couldn't reach far enough back but that I can better now’* |
| Delivery of CBE | The model of delivery was generally considered to be appropriate, however, doing them more frequently and for longer was welcomed. | *‘…was good but we didn't do it long enough I don’t think’* |
|  |  | *‘I think we could do it longer*  *automatically we ought to do it most weeks’* |
| Barriers to participation | Barriers to participation focused around poor health status and limited physical abilities. | *'I was feeling ill all the time'* |
|  |  | *'I had a chest infection during the time and these things pull you down'* |
|  |  | *‘I'm not sure more people could have done it- some are very poorly One has a bad chest* |
| Motivators to participation | Participants commented on the support and encouragement of the group to help motivate them to take part. The characteristic and support from the exercise instructor was also discussed in relation to supporting participation. | *'When I saw the others I thought come on get on with it’* |
|  |  | *'If there were two of you a minimum of two you have to try not to let yourself down’* |
|  |  | *‘The lady was lovely*  *the lady was very good- and if we got it wrong she didn't mind, she explained'* |

Table 4: Summary of views of staff

|  | Summary | Supporting quotes |
| --- | --- | --- |
| Perceptions of CBE | Discussions focused on chair based exercise being appropriate due to safety and physical limitations. Staff in centres where some seated exercise programmes were already delivered found the study CBE intervention to be more professional and calmer. | *‘when you say chair based exercise you know it’s not going to be too energetic’* |
|  |  | *‘I think it is better to sit in a chair and do something- you don't want them falling over and things like that’* |
|  |  | *‘I think it’s a very good idea, I think the whole process of being safe in a chair is very good and its very popular*  *it’s really the only exercises they can do’* |
|  |  | *‘I do think the chair based exercises was a lot calmer [than other physical activities at the centre], controlled movement’* |
| Benefits of CBE | Staff observed social and well-being benefits of the intervention, however, physical benefits were not commonly discussed. | *‘…as the weeks went past he definitely became more active and wanted to come in’* |
|  |  | *‘…yeah they talked more and I noticed 2 of them they do chat a lot more than wat they probably would have done;* |
|  |  | *‘I thought sometimes when she had done, you know the exercises her mobility seemed better’* |
|  |  | *‘..as in themselves when they came back into the group room they were a lot happier and enjoyed it, very talkative’* |
| Delivery of CBE | The delivery of the CBE intervention was difficult for staff to comment on as they had not all been involved. One activity coordinator did think the programme was too intense for the participants. One staff member felt the target group for the programme needed to be much younger and perhaps it was too little too late. | *‘I think the exercise was quite intense… I mean I did I had some sessions where I was aching the next day in my shoulder’ [staff member who joined in the programme at the centre]* |
|  |  | *‘I think it was too much too late, I think the residents you were working on are very elderly’* |
| Barriers to participation | Barriers to participation focused around poor health status and limited physical abilities. | *‘…no it’s hard because obviously with mobility and like I say there’s different people have do different mobile needs’* |
|  |  | *‘…it’s hard for them having the illness that they've got to understand what’s happening*  *just not very well’* |
| Motivators to participation | Staff comments that older people often require motivation and support from staff as well as observing others. | *‘…she saw everyone else joining in and she thought mmm I'm missing out.* |
|  |  | *.But of course some people if you don't encourage them will just sit there and give up.* |
|  |  | *‘…people that were quite adamant they were not going to do activities absolutely loved this programme, I was watching thinking ‘you wouldn't do that for me, you won't come out of your room and exercise with me’ … means I have made friends with some people that perhaps were very reticent to come forward and do anything.* |
